# Effect of Implantable Collamer Lens on Anterior Segment Measurement and Intraocular Lens Power Calculation Based on IOLMaster 700 and Sirius

**DOI:** 10.1155/2021/8988479

**Published:** 2021-12-21

**Authors:** Xiaoyong Chen, Di Zhang, Ziyuan Liu, Yinan Liu, Hongyuan Cai, Qianru Wu, Yu Zhang

**Affiliations:** ^1^Department of Ophthalmology, Peking University Third Hospital, 49 North Garden Road, Haidian District, Beijing 100191, China; ^2^Beijing Key Laboratory of Restoration of Damaged Ocular Nerve, Peking University Third Hospital, 49 North Garden Road, Haidian District, Beijing 100191, China; ^3^Beijing Tongren Eye Center, Beijing Tongren Hospital, Beijing Institute of Ophthalmology, Capital Medical University, Beijing 100730, China

## Abstract

**Purpose:**

To investigate the possible effect of an implantable collamer lens (ICL) on ocular biometrics and intraocular lens (IOL) power calculation.

**Methods:**

Ocular measurements were taken preoperatively and at the two-month follow-up using IOLMaster 700 and Sirius in 85 eyes (43 patients) who had previously undergone ICL surgery. IOL power was calculated using either IOLMaster 700 (Barrett Universal II formula) or Sirius (ray-tracing). All data were compared using the paired *t*-test.

**Results:**

The difference between preoperative and postoperative anterior chamber depth (ACD), lens thickness (LT), and keratometry on the steep axis (K2) measured by IOLMaster 700 was statistically significant (*p* < 0.001). In 11 of 85 eyes, IOLMaster misjudged the anterior surface of the ICL as that of the lens, leading to an error in ACD and LT. There were no significant differences between preoperative and postoperative axial length (AL) (*p* = 0.223), white to white (WTW) (*p* = 0.100), keratometry on flat axis (K1) (*p* = 0.117), or central corneal thickness (CCT) (*p* = 0.648), measured using IOLMaster. The difference in IOL power calculated using the Barrett II formula was significant (*p* = 0.013). Regression analysis showed that AL and K had the greatest influence on IOL calculation (*p* < 0.001), and ACD and LT had less influence (*p* = 0.002, *p* = 0.218, respectively). K1 and K2 were modified to exclude the influence of K2, and modified IOLs showed no difference between pre and postoperation (*p* = 0.372). Preoperative and postoperative ACD measured using Sirius were significantly different (*p* < 0.001); however, the IOL power calculated using ray-tracing technology showed no significant differences (*p* > 0.05).

**Conclusions:**

The ocular biometric apparatus may misjudge the anterior surface of the lens, resulting in measurement errors of ACD and LT, which has little effect on the calculation of IOL power when using IOLMaster 700 (Barrett Universal II formula) and Sirius (ray-tracing).

## 1. Introduction

Implantable collamer lenses (ICLs) are gaining popularity for correcting myopia due to their good visual acuity [1], predictability [2], and reversibility [3]; however, previous literature reported anterior subcapsular cataract as a postoperative complication of ICL, which needed to be extracted with ICL removal and intraocular lens (IOL) implantation [4, 5], though the reported incidence of anterior subcapsular cataract with the V4c ICL is vanishingly small (Packer M., 2018). In addition, due to the widespread use of ICL globally, a large number of patients with ICL will develop age-related cataracts and require cataract surgery in the future [6]. Ocular measurements and IOL calculations are crucial for the refractive outcome of cataract surgery. This is especially true for ICL patients with high expectations of spectacle-free status postoperatively.

The influence of ICL on ocular measurements and IOL calculation has already been reported by other authors. Amro et al. analyzed the effect of ICL on biometry using the IOLMaster 500 and IOL calculation with third- and fourth-generation formulas [7]. Other authors studied the axial length (AL) alterations before and after phakic intraocular lens implantation with IOLMaster 500 or older versions IOLMaster [8–10].

The methods of ocular biometrics have evolved rapidly. Swept-source optical coherence tomography (SS-OCT), represented by IOLMaster 700, will gradually replace partial coherence interferometry (PCI) of IOLMaster 500. In addition, the Scheimpflug/Placido imaging system represented by Sirius and Pentacam AXL has clinical advantages and is widely used. As for IOL calculation, despite the theoretical formulas are commonly used in calculations of IOL power, the accuracy of the ray-tracing technique has been reported previously [11]. It remains unknown whether the new apparatus and technology are affected by ICL implantation. In this study, we analyzed the changes in anterior segment measurement and IOL power calculation using IOLMaster 700 (Barrett Universal II formula) and Sirius (ray-tracing) before and three months after ICL implantation.

## 2. Materials and Methods

Between January 2021 and March 2021, 43 patients (85 eyes) were enrolled at the Department of Ophthalmology, Peking University Third Hospital. All patients met the indications for ICL implantation surgery and signed an informed consent agreement [12]. Exclusion criteria included corneal leucoma, fundus diseases, and any other eye diseases that may affect the accuracy of the ocular biometric parameters. Patients with astigmatism more than 1.5 D were implanted with the spherocylindrical ICL, and the others underwent spherical ICL implantation surgeries. All patients were followed up for at least two months postoperatively. This study adhered to the tenets of the Declaration of Helsinki and was approved by the Ethics Committee of Peking University Third Hospital.

Preoperative and postoperative eye biometric measurements at the 3-month follow-up were conducted using the IOLMaster 700 (Carl Zeiss Meditec; Jena, Germany), Sirius (CSO; Florence, Italy), and the anterior segment optical coherence topography (AS-OCT) system (Tomey OA-2000; Tomey Inc., Japan). The IOLMaster 700 is a swept-source OCT, which uses a 1055 nm laser source to measure the anterior segment parameters and axial length [13, 14]. Sirius uses the Scheimpflug/Placido imaging principle to establish a three-dimensional panorama of the anterior segment from the corneal epithelium to the posterior capsule of the lens, which includes a rotating Scheimpflug camera, and can capture photos of 25000 pixel in 2 s [15, 16]. AS-OCT is also a swept-source OCT which can directly measure the anterior chamber depth as a reference. All patients were measured in a dark room with the natural pupil size.

All ICL implantation surgeries were performed by a single experienced surgeon (Y. Z.). All patients were given dilating and cyclopegic agents 1 h before surgery. A topical anesthetic of one drop of 0.4% oxybuprocaine hydrochloride eye drops (Benoxil, Santen; Osaka, Japan) was administered three times every 5 min before surgery. A 3.0 mm clear corneal incision at the steepest meridian and 0.8 mm subsidiary incision 90° anticlockwise were made. After injecting the viscoelastic agent into the anterior chamber, the ICL (V4c; STAAR Surgical, Switzerland) was inserted into the anterior chamber. The ICL was then placed into the posterior chamber by adjusting the loops, and the remaining viscoelastic agent was completely washed out with a balanced salt solution.

### 2.1. Statistical Analysis

Statistical analysis was performed using SPSS (version 22.0; IBM SPSS Statistics, Armonk, NY, USA). All data are presented as the mean ± standard deviation (SD). The difference between preoperative and postoperative data was analyzed by the paired *t*-test, and linear regression analysis was used to identify the effect of each parameter on the IOL calculation. Statistical significance was set at *p* < 0.05.

## 3. Results

This study included 85 eyes (total, 43 patients; 15 men and 28 women) who underwent ICL V4c implantation surgery. There were 22 eyes implanted with the spherocylindrical ICL. The mean age was 27.19 ± 6.20 years old. The preoperative spherical equivalent (SE) was −7.77 ± 2.28D (ranging from −2.75D to −12.25D) and the postoperative SE was +0.15 ± 0.24D (ranging from + 0.75D to −0.50D). The mean cylinder diopter of preoperative patients was −1.26 ± 0.99D and that of postoperative patients was −0.28 ± 0.32D. The average best corrected visual acuity (logMAR units) before surgeries was 0.012 ± 0.032 and the uncorrected visual acuity (logMAR units) 2 months after surgeries was −0.069 ± 0.052. There were no complications during the surgery or follow-up period.

### 3.1. IOLMaster Measurement of Anterior Segment Parameters after ICL Implantation

**Figure 1:**
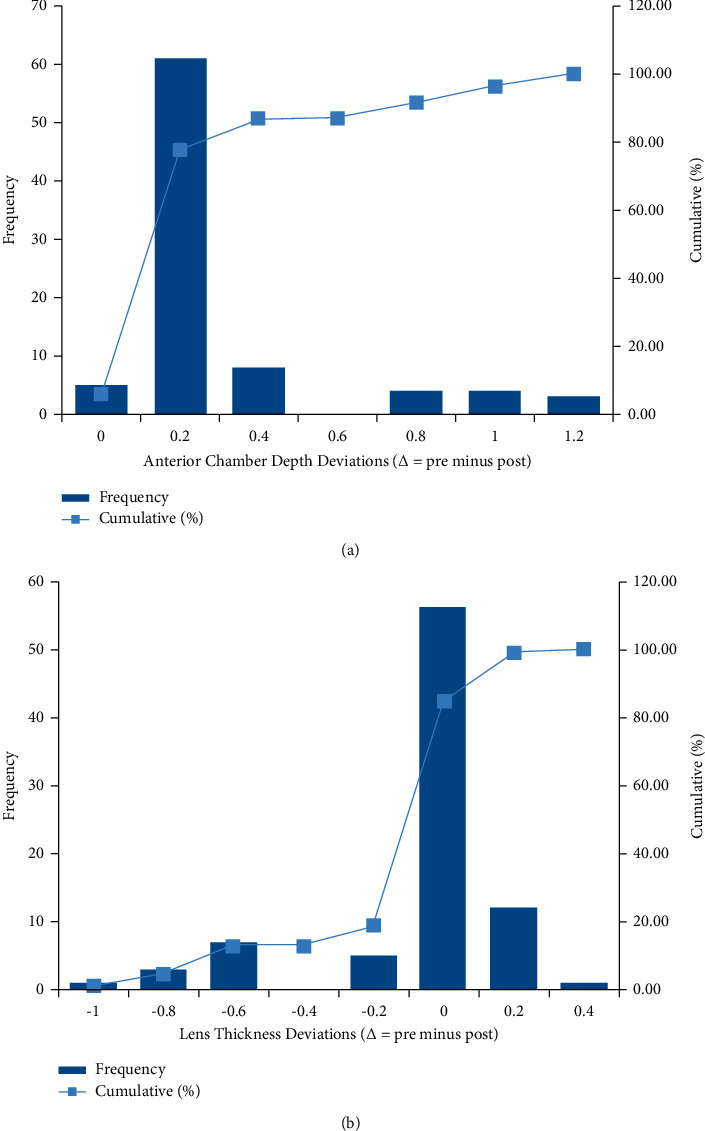
(a) The distribution of preoperative and postoperative anterior chamber depth deviations (∆ = pre minus post). (b) The distribution of preoperative and postoperative lens thickness deviations (∆ = pre minus post).

**Figure 2:**
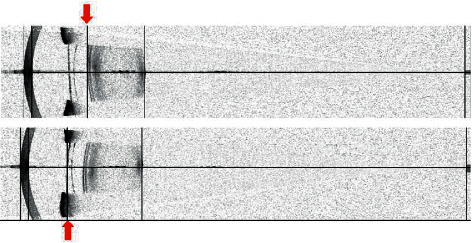
The IOLMaster images of a 28 year-old woman. The anterior surface of the right eye's lens (above) was accurately identified by IOLMaster, while in the left eye (below), the anterior surface of ICL was mistaken as the anterior surface of lens.

The preoperative ACD (3.69 ± 0.23 mm) and the postoperative ACD (3.49 ± 0.38 mm) were significantly different (*p* < 0.001) (Table 1). Deviations of ACD (∆ = pre minus post) ranged from −0.04 mm to 1.13 mm. About 74 eyes (87.06%) showed ACD deviations of less than 0.3 mm, and 11 eyes (12.94%) showed ACD deviations of more than 0.6 mm (Figure 1(a)). Further analysis of the measurements of these 11 eyes revealed that the IOLMaster mistook the anterior surface of the ICL for the anterior surface of the lens and thus had shallower postoperative ACD (Figure 2). Interestingly, after removing the mismeasured 11 eyes from the sample, the ACDs before and after surgery were also significantly different (3.71 ± 0.22 mm and 3.61 ± 0.22 mm, respectively; *p* < 0.001) (Table 2).

The difference in the preoperative LT (3.59 ± 0.22 mm) and postoperative LT (3.75 ± 0.35 mm) was statistically significant (*p* < 0.001) (Table 1). The deviation (∆ = pre minus post) ranged from −1.02 mm to 0.27 mm, and 11 eyes (12.94%) were less than −0.6 mm, which was consistent with the eyes whose ACD values were mismeasured (Figure 1(b)).

There was no significant difference between preoperative and postoperative AL (*p* = 0.223), WTW (*p* = 0.100), corneal flat axis curvature K1 (*p* = 0.117), and CCT (*p* = 0.648). However, the difference between preoperative and postoperative corneal steep axis curvature K2 was statistically significant, which was possibly caused by the corneal incision made at the steep axis (*p* < 0.001) (Table 1).

### 3.2. IOL Power Calculation by Barrett Universal II Formula

The preoperative and postoperative biometric parameters of 85 eyes were entered into the Barrett Universal II formula to calculate the IOL power. When the target diopter was set at 0, the preoperative IOL power was 12.54 ± 2.69 D, and the postoperative IOL power was 12.62 ± 2.62 D (*p* = 0.013). The median absolute deviation was 0.20 D, and the average absolute deviation was 0.22 D. The deviation range was from −0.69 D to 0.83 D. Seventy-nine eyes (92.94%) had a deviation within ± 0.5 D, of which 52 eyes (61.18%) had a deviation within ± 0.25 D and 6 eyes (7.06%) had a deviation beyond ± 0.5 D.

The 11 eyes with wrongly recognized anterior surface of the lens were removed, and the remaining 74 eyes were entered into the Barrett Universal II formula to calculate the IOL power. When the target diopter was set as 0, the preoperative IOL power was 12.46 ± 2.68 D and the postoperative IOL power was 12.55 ± 2.60 D (*p* = 0.004). The preoperative IOL power for those 11 eyes was 13.12 ± 2.85 D, and the postoperative IOL power was 13.06 ± 2.81 D (*p* = 0.328).

Linear regression analysis was used to identify the effect of each parameter on the calculation of IOL. The deviation of preoperative and postoperative IOL power was set as the dependent variable, and the deviation of preoperative and postoperative ACD, LT, AL, K1, K2, and WTW were set as independent variables. The *R*^2^ was 94.8%, and the standardized beta coefficient is given in Table 3. The LT difference had the least effect (*p* = 0.218), while AL and corneal curvature had the greatest effect (*p* < 0.001). ACD and WTW also showed a noticeable impact (*p* = 0.002 and *p* = 0.015, respectively).

Since the significant change in K2 caused by corneal incision in surgeries interfered strongly with IOL calculation, we applied preoperative K1 and K2 and postoperative ACD, LT, AL, and WTW to Barrett Universal II formula to obtain a modified IOL power (12.53 ± 2.67 D). When compared with preoperative IOL power, no significant difference was found (*p* = 0.372). A similar result was obtained after the wrongly measured 11 eyes were removed (12.46 ± 2.65 D, *p* = 0.976). The significant difference in IOL power seen before and after surgery was mostly derived from the changes in K2 instead of the deviation of the ACD and LT measurements, though this change in IOL calculation is not erroneous.

### 3.3. Anterior Segment Parameters Measurements and IOL Calculation with Sirius

The anterior segment parameters measured by Sirius before and three months after ICL implantation are given in Table 4. There was a significant difference in the ACD (*p* < 0.001). The postoperative ACD was significantly lower than the preoperative, and the deviation range of ACD was 0.28 mm–1.57 mm.

Since Sirius cannot display the image of the specific measurement position, we also conducted AS-OCT to explore whether the ACD difference by Sirius was derived from the misidentification of the lens. It was found that the postoperative ACD measured by Sirius showed no significant difference in regards to the distance from the corneal endothelium to the anterior surface of ICL (2.30 ± 0.28 mm; *p* = 0.460), while a significant difference regarding the distance from the endothelium to the front surface of the lens was seen (2.54 ± 0.27 mm; *p* < 0.001). This suggests that Sirius might mistakenly identify the anterior surface of the ICL as the anterior surface of the lens when measuring the ACD.

Additionally, the IOL power was calculated using the ray-tracing technology. The IOL power with the target diopter closest to 0 was selected, the average of which was 11.49 ± 2.93 D preoperatively and 11.43 ± 2.85 D postoperatively (Table 4). The difference found was not statistically significant (*p* > 0.05). The median absolute deviation of the IOL power was 0.5 D, and the mean absolute deviation was 0.56 D. The deviation range was from −2.00 D to 1.50 D. The deviation was beyond ± 0.5 D in 18 eyes (21.18%) and beyond ± 1.0 D in 6 eyes (7.01%).

## 4. Discussion

Accurate preoperative measurements are very important in refractive cataract surgery [13]. At present, there are a variety of advanced optical biometry instruments used in clinics, including IOLMaster 700, IOLMaster 500, Pentacam AXL, and Sirius, and each use different methods to measure axial length and anterior segment parameters [14–16]. Different measurement methods may be affected by ICL, resulting in data deviation and inaccurate calculations of IOL power. However, with few related studies, there is no consistent conclusion at present. ICL implantation surgery involves placing the ICL into the ciliary sulcus through a corneal incision [17]. The main part of the ICL material is hydrophilic carboxymethyl acrylate combined with 0.14% collagen copolymer, with a water content as high as 40%, resulting in high light transmittance and softness [18, 19]. Whether the existence of ICL in the light pathway may alter anterior segment measurement using an optical measuring device is unknown.

In this study, IOLMaster 700 was used to measure AL pre and postoperation, with the resulting difference being not statistically significant. In addition, there were no significant differences in CCT, WTW, and K1. The obvious change in corneal K2 may be related to the incision at the steep axis. Elmohamady et al. found that there was no significant difference in central corneal thickness, pupil diameter, and keratometry between pre and postoperative eyes [20]. Yu Ayong et al. used IOLMaster 500 to measure the difference in AL before and after ICL operation and found that the difference was not more than 0.1 mm. According to the SRK formula, the error of IOL degree caused by this AL error was no more than 0.25 D, which had no obvious clinical significance [9]. It is accepted that AL measured by SS-OCT has more accuracy than that by PCI which is used in IOLMaster 500 [21]. Some authors suggest that SS-OCT is the gold standard for AL measurement [22]. In the medium-long eyes, especially, the predictive accuracy of SS-OCT for IOL calculations was higher than PCI [23]. However, it is known that the SS-OCT biometer with group refractive index (IOLMaster 700) overestimates AL compared to the device providing segmented AL in the long eyes, and Cooke-modified AL (CMAL) is the adjusting method that gives the AL values closest to the segmented axial length [24]. In our research, we used the self-control method which compared one patient's preoperative and postoperative data, and thus, we considered that adjusted AL was not a must in this study.

This study also focused on the differences in ACD and LT before and after ICL implantation. We found that the ACD deviation of a small number of patients was significantly higher than that of the other patients. This difference was caused by the instrument mistakenly recognizing the anterior surface of the ICL as the anterior surface of the lens. At the same time, the LT of these patients also showed obvious measurement deviations. Even if the ICL special mode of IOLMaster was used, the front surface of the ICL may be mistakenly identified as the front surface of the lens, resulting in measurement errors. In the patients whose anterior lens surface was correctly identified by the instrument, there was still a statistical difference in ACD and LT before and after the operation. It seemed that the ICL might alter the light source during the measurement procedure.

Then, the second question emerged. Will this deviation in ACD and LT influence IOL calculation? Most of the ACD deviations were less than 0.3 mm. It was reported that the deviation of ACD within 0.2 mm would cause the deviation of IOL within 0.1 *D*, which had little clinical significance [25]. Regression analysis showed that the difference in IOL power was mainly affected by keratometry and AL, but less affected by ACD and LT. Additionally, when modifying the K values, no significant difference between preoperative IOL and postoperative IOL was seen. The measuring deviations of ACD and LT did not affect the IOL calculation, which meant that less focus was needed to identify the lens interface when taking measurements.

The ACD measured by Sirius after ICL implantation was significantly lower than that before surgery. Sirius had no image showing the measured position; therefore, AS-OCT measurements were also conducted. It was found that the ACD measured by Sirius showed no statistical difference regarding the distance from the endothelium to the anterior surface of the ICL measured by AS-OCT, but a statistically significant difference was seen regarding the distance from the endothelium to the front surface of the lens. The results suggest that Sirius might not distinguish ICL from the natural lens, or Sirius might take the iris plate as a reference when measuring ACD resulting in the aforementioned differences. However, it had no significant impact on Sirius' ray-tracing calculation of IOL power in this study. It is still unknown whether the measurements of the Scheimpflug/Placido imaging system used in the theoretical formula would work on the ICL eyes.

Our study had some limitations: (1) the sample size was small; (2) all patients had clear lenses—whether lens opacity will affect the accuracy of ocular biometrics remains to be explored; (3) the application of other instruments capable of calculating the power of the intraocular lens, such as Pentacam AXL, still needs to be further explored for the biometry and calculation of IOL power; (4) the influence of K alterations on the IOL calculation could not be avoided in this study; (5) the accuracy of IOL calculations of ICL patients who undergo cataract extraction, ICL removal, and IOL implantation will need to be analyzed further.

## 5. Conclusions

In conclusion, the common ocular biometric apparatus IOLMaster 700 may misjudge the anterior surface of the lens in the eyes implanted with ICL, resulting in measurement errors of the ACD and LT. However, such a mismeasurement does not affect the calculation of IOL power when entering IOLMaster data into the Barrett Universal II formula. ICL may lead to significant deviations in the measurement of ACD using Sirius, but it does not affect the calculation of the IOL degree by the ray-tracing method.

## Figures and Tables

**Table 1:** The biometric parameters measured by IOLMaster.

Parameters	Preoperative	3-month postoperative	*P*
ACD (mm)	3.69 ± 0.23	3.49 ± 0.38	< 0.001^*∗*^
LT (mm)	3.59 ± 0.22	3.75 ± 0.35	< 0.001^*∗*^
AL (mm)	26.21 ± 1.23	26.20 ± 1.22	0.233
WTW (mm)	11.86 ± 0.38	11.88 ± 0.40	0.100
K1 (D)	43.27 ± 1.24	43.31 ± 0.14	0.117
K2 (D)	44.71 ± 1.62	44.54 ± 1.66	< 0.001^*∗*^
CCT (*μ*m)	516.09 ± 30.51	515.69 ± 31.14	0.648
IOL (D)	12.54 ± 2.69	12.61 ± 2.62	0.013^*∗*^

ACD, anterior chamber depth; LT, lens thickness; AL, axial length; WTW, white to white; K1, corneal flat axis curvature; K2, corneal steep axis curvature; CCT, central corneal thickness; IOL, intraocular lens. ^*∗*^*P* < 0.05.

**Table 2:** The biometric parameters measured by IOLMaster excluding 11 misjudged eyes.

Parameters	Preoperative	3-month postoperative	*P*
ACD (mm)	3.71 ± 0.22	3.61 ± 0.22	< 0.001^*∗*^
LT (mm)	3.58 ± 0.23	3.64 ± 0.22	< 0.001^*∗*^
AL (mm)	26.27 ± 1.21	26.26 ± 1.20	0.310
WTW (mm)	11.86 ± 0.37	11.89 ± 0.40	0.038^*∗*^
K1 (D)	43.20 ± 1.14	43.25 ± 1.17	0.082
K2 (D)	44.64 ± 1.61	44.46 ± 1.64	< 0.001^*∗*^
CCT (*μ*m)	518.93 ± 28.96	517.91 ± 30.26	0.271
IOL (D)	12.46 ± 2.68	12.55 ± 2.60	0.004^*∗*^

ACD, anterior chamber depth; LT, lens thickness; AL, axial length; WTW, white to white; K1, corneal flat axis curvature; K2, corneal steep axis curvature; CCT, central corneal thickness; IOL, intraocular lens. ^*∗*^*P* < 0.05.

**Table 3:** Regression analysis of preoperative and postoperative IOL power difference.

Values	ACD deviation	LT deviation	AL deviation	K1 deviation	K2 deviation	WTW deviation
Standard coefficient beta	0.287	0.108	−0.504	−0.561	−0.652	0.066
*P*	0.002^*∗*^	0.218	< 0.001^*∗*^	< 0.001^*∗*^	< 0.001^*∗*^	0.015^*∗*^

ACD, anterior chamber depth; LT, lens thickness; AL, axial length; WTW, white to white; K1, corneal flat axis curvature; K2, corneal steep axis curvature. ^*∗*^*P* < 0.05.

**Table 4:** Sirius measurements of anterior segment before and after ICL implantation.

Parameters	Preoperative	3-month postoperative	*P*
ACD (mm)	3.26 ± 0.23	2.32 ± 0.28	< 0.001^*∗*^
CCT (*μ*m)	520.58 ± 31.93	520.47 ± 32.32	0.872
K1 (D)	43.18 ± 1.26	43.28 ± 1.31	0.057
K2 (D)	44.50 ± 1.70	44.40 ± 1.76	0.072
IOL (D)	11.49 ± 2.93	11.43 ± 2.85	0.653

ACD, anterior chamber depth; CCT, central corneal thickness; K1, K on flat axis; K2, K on steep axis. ^*∗*^*P* < 0.05.

## Data Availability

The datasets used and analyzed during the current study are available from the corresponding author on reasonable request.

## References

[1] Alfonso J. F., Lisa C., Alfonso-Bartolozzi B., Pérez-Vives C., Montés-Micó R. (2014). Collagen copolymer toric phakic intraocular lens for myopic astigmatism: one-year follow-up. *Journal of Cataract & Refractive Surgery*.

[2] Bhandari V., Karandikar S., Reddy J. K., Relekar K. (2015). Implantable collamer lens V4b and V4c for correction of high myopia. *Journal of Current Ophthalmology*.

[3] Zhang J., Shao J., Zheng L., Zhao X., Sun Y. (2021). Changes in ocular parameters the crystalline lens after implantation of a collamer lens. *Clinical and Experimental Optometry*.

[4] Kocová H., Vlková E., Michalcová L., Rybárová N., Motyka O. (2017). Incidence of cataract following implantation of a posterior-chamber phakic lens ICL (Implantable Collamer Lens) - long-term results. *Casopis Ceske Oftalmologicke Spolecnosti a Slovenske Oftalmologicke Spolecnosti*.

[5] Gimbel H. V., LeClair B. M., Jabo B., Marzouk H. (2018). Incidence of implantable Collamer lens-induced cataract. *Canadian Journal of Ophthalmology*.

[6] Pinto C., Monteiro T., Franqueira N., Faria-Correia F., Mendes J., Vaz F. (2021). Posterior chamber collamer phakic intraocular lens implantation: comparison of efficacy and safety for low and moderate-to-high myopia. *European Journal of Ophthalmology*.

[7] Amro M., Chanbour W., Arej N., Jarade E. (2018). Third- and fourth-generation formulas for intraocular lens power calculation before and after phakic intraocular lens insertion in high myopia. *Journal of Cataract & Refractive Surgery*.

[8] Sanders D. R., Bernitsky D. A., Harton P. J., Rivera R. R. (2008). The Visian myopic implantable collamer lens does not significantly affect axial length measurement with the IOLMaster. *Journal of Refractive Surgery (Thorofare, N.J.: 1995)*.

[9] Yu A., Wang Q., Zhu S., Xue A., Su Y., Pan R. (2015). Effects of posterior chamber phakic intraocular lens on axial length measurements. *Zhonghua Yan Ke Za Zhi*.

[10] Wang Z. Y., Yang W. L., Li D. J. (2019). Comparison of biometry with the Pentacam AXL, IOLMaster 700 and IOLMaster 500 in cataract patients. *Zhonghua Yan Ke Za Zhi*.

[11] Gjerdrum B., Gundersen K. G., Lundmark P. O., Aakre B. M. (2021). Refractive precision of ray tracing IOL calculations based on OCT data versus traditional IOL calculation formulas based on reflectometry in patients with a history of laser vision correction for myopia. *Clinical Ophthalmology*.

[12] Diaz-Llopis M., Montero J., Amselem L., Udaondo P., Garcia-Delpech S. (2008). Posterior chamber phakic intraocular lenses: a comparative study between ICL and PRL models. Choosing/selection criteria. *Archivos de la Sociedad Espanola de Oftalmologia*.

[13] Bullimore M. A., Slade S., Yoo P., Otani T. (2019). An evaluation of the IOLMaster 700. *Eye and Contact Lens: Science and Clinical Practice*.

[14] Chan T. C. Y., Wan K. H., Tang F. Y., Wang Y. M., Yu M., Cheung C. (2020). Repeatability and agreement of a swept-source optical coherence tomography-based biometer IOLMaster 700 versus a Scheimpflug imaging-based biometer AL-scan in cataract patients. *Eye and Contact Lens: Science and Clinical Practice*.

[15] Fan R., Chan T. C., Prakash G., Jhanji V. (2018). Applications of corneal topography and tomography: a review. *Clinical and Experimental Ophthalmology*.

[16] Pereira J. M. M., Neves A., Alfaiate P., Santos M., Aragão H., Sousa J. C. (2018). Lenstar LS 900 vs Pentacam-AXL: comparative study of ocular biometric measurements and intraocular lens power calculation. *European Journal of Ophthalmology*.

[17] Chen P., Cai X., Xu L. (2017). Assessing oxygen saturation in retinal vessels in high myopia patients pre- and post-implantable collamer lens implantation surgery. *Acta Ophthalmologica*.

[18] Martin R. G., Sanders D. R. (2005). A comparison of higher order aberrations following implantation of four foldable intraocular lens designs. *Journal of Refractive Surgery*.

[19] Nakamura T., Isogai N., Kojima T. (2020). Long-term in vivo stability of posterior chamber phakic intraocular lens: properties and light transmission characteristics of explants. *American Journal of Ophthalmology*.

[20] Elmohamady M. N., Abdelghaffar W. (2017). Anterior chamber changes after implantable collamer lens implantation in high myopia using Pentacam: a prospective study. *Ophthalmology and Therapy*.

[21] Yang C. M., Lim D. H., Kim H. J., Chung T.-Y. (2019). Comparison of two swept-source optical coherence tomography biometers and a partial coherence interferometer. *PLoS One*.

[22] Huang J., Chen H., Li Y. (2019). Comprehensive comparison of axial length measurement with three swept-source OCT-based biometers and partial coherence interferometry. *Journal of Refractive Surgery*.

[23] Whang W.-J., Yoo Y.-S., Kang M.-J., Joo C.-K. (2018). Predictive accuracy of partial coherence interferometry and swept-source optical coherence tomography for intraocular lens power calculation. *Scientific Reports*.

[24] Savini G., Hoffer K. J., Carballo L., Taroni L., Schiano-Lomoriello D. (2021). A comparison of different methods to calculate the axial length measured by optical biometry. *Journal of Cataract & Refractive Surgery*.

[25] Lackner B., Schmidinger G., Skorpik C. (2005). Validity and repeatability of anterior chamber depth measurements with Pentacam and Orbscan. *Optometry and Vision Science*.

